# Evaluation of the Carba NP test for carbapenemase detection in Enterobacteriaceae, *Pseudomonas* spp. and *Acinetobacter* spp., and its practical use in the routine work of a national reference laboratory for susceptibility testing

**DOI:** 10.1007/s10096-017-3062-0

**Published:** 2017-07-25

**Authors:** E. Literacka, M. Herda, A. Baraniak, D. Żabicka, W. Hryniewicz, A. Skoczyńska, M. Gniadkowski

**Affiliations:** 1 Department of Epidemiology and Clinical Microbiology, National Medicines Institute, ul. Chełmska 30/34, 00-725 Warsaw, Poland; 2 Department of Molecular Microbiology, National Medicines Institute, ul. Chełmska 30/34, 00-725 Warsaw, Poland

## Abstract

The aim of this study was to evaluate the Carba NP test (and CarbAcineto) for the detection of carbapenemases in Enterobacteriaceae, *Pseudomonas* spp. and *Acinetobacter* spp., and to assess its usefulness in the routine work of the National Reference Centre for Susceptibility Testing (NRCST) in Poland. The evaluation of the Carba NP/CarbAcineto tests was carried out on a group of 81 Enterobacteriaceae, *Pseudomonas* spp. and *Acinetobacter* spp. isolates producing KPC-, NDM-, VIM-, IMP- or OXA-48, -23, -24/40, -58-type carbapenemases, and on 26 carbapenemase-negative strains cultivated on a broad panel of microbiological media. Subsequently, the performance of the Carba NP/CarbAcineto tests was assessed on 1282 isolates of Enterobacteriaceae, *Pseudomonas* spp. and *Acinetobacter* spp. from Polish hospitals, submitted to the NRCST during a 9-month period in 2014. The Carba NP/CarbAcineto results were compared with other phenotypic tests and/or polymerase chain reaction (PCR). The impact of the media on the results of the Carba NP/CarbAcineto tests was observed, with the Columbia blood agar yielding the highest sensitivity and clarity of the results. Furthermore, the Carba NP/CarbAcineto tests were included in the NRCST routine procedure for carbapenemase identification. The sensitivity and specificity of the Carba NP test were 95.8% and 93.3%, respectively, for Enterobacteriaceae, and 97.5% and 99.0%, respectively, for *Pseudomonas* spp. The sensitivity of the CarbAcineto test for *Acinetobacter* spp. was 88.9%. This study confirmed the usefulness of the Carba NP/CarbAcineto tests for the rapid detection of various types of carbapenemases.

## Introduction

The dynamic spread of carbapenemase-producing Enterobacteriaceae, *Pseudomonas* spp. and *Acinetobacter* spp. has been observed all over the world in recent years. The predominant carbapenemases include β-lactamases of the Ambler class A (KPCs), metallo-β-lactamases (MBLs) of the class B (VIMs, IMPs, NDMs) and carbapenem-hydrolysing oxacillinases of the class D (OXA-23, OXA-24/40, OXA-48 or OXA-58 types) [1–9]. Carbapenemase producers notoriously exhibit multidrug resistance phenotypes, making treatment of infections extremely difficult [10–13]. Therefore, quick, unambiguous, easy and cheap identification of carbapenemase-producing bacteria has been one of the priorities of modern clinical microbiology. The biochemical Carba NP test for Enterobacteriaceae and *Pseudomonas* spp., and its variant CarbAcineto for *Acinetobacter* spp., were proposed to address this challenge in 2012 and 2014, respectively [1, 14, 15]. Both tests were reported to have high specificity and sensitivity for all of the major carbapenemase types [1, 14, 15]; however, it was shown that, for some enzymes, the sensitivity relies strongly on the type of microbial medium used for bacteria cultivation [16–18]. The original Carba NP test was soon followed by modified variants, and has been commercialised [19–26].

The aim of this study was to evaluate the Carba NP and CarbAcineto tests for the detection of carbapenemase-producing Enterobacteriaceae, *Pseudomonas* spp. and *Acinetobacter* spp. cultivated on different media. Moreover, the Carba NP/CarbAcineto tests were assessed in the routine diagnostics of carbapenemase producers conducted by the National Reference Centre for Susceptibility Testing (NRCST) in Poland in 2014.

## Materials and methods

### Assessment of the Carba NP and CarbAcineto tests: strain collection and cultivation media

For the initial evaluation of the Carba NP and CarbAcineto tests, a total of 107 strains were used: 81 carbapenemase producers that were well characterised with phenotypic methods, polymerase chain reaction (PCR) and sequencing, and 26 carbapenemase non-producers, including 21 meropenem-non-susceptible strains (Table 1). Both tests were performed by the in-house protocol as previously described [14, 18]. The results presented are those that were recorded after 2 h of incubation time. For cultivation of bacteria, a number of non-selective or screening media and the Mueller–Hinton ready-to-use agar plates, products of six manufacturers, were used (Tables 2 and 3).

**Table 1:** Isolates used in the Carba NP/CarbAcineto tests evaluation

Species, total number	Carbapenemase genes	No.
Carbapenemase-positive, *n* = 81		
Enterobacteriaceae, *n* = 34		
*K. pneumoniae*, *n* = 15	KPC	4
	NDM	5
	VIM	2
	IMP	1
	OXA-48	3
*K. oxytoca*, *n* = 3	NDM	1
	VIM	2
*E. coli*, *n* = 9	KPC	2
	NDM	4
	OXA-48	3
*E. cloacae*, *n* = 5	KPC	1
	NDM	1
	VIM	3
*C. freundii*, *n* = 2	KPC	2
*Pseudomonas* spp., *n* = 15		
*P. aeruginosa*, *n* = 9	VIM	8
	IMP	1
*P. putida*, *n* = 6	VIM	6
*Acinetobacter* spp., *n* = 32		
*A. baumannii*	VIM	6
*A. lwoffii*	VIM	7
*A. junii*	VIM	3
*A. baumannii*	OXA-23	9
*A. baumannii*	OXA-24/40	6
*A. baumannii*	OXA-58	1
Carbapenemase-negative, *n* = 26^a^		
*K. pneumoniae*	No	10
*P. aeruginosa*	No	6
*Acinetobacter* spp*.*	No	10

^a^Of the 26 carbapenemase-negative isolates, 21 isolates were carbapenem-non-susceptible (8 *K. pneumoniae*, 6 *P. aeruginosa* and 7 *Acinetobacter* spp.)

**Table 2:** The results of the Carba NP test for Enterobacteriaceae isolates (34 carbapenemase producers and 10 carbapenemase non-producers) cultivated on a set of microbial media

Microbiological media	Manufacturer	VIM; *n* = 7^a^				IMP; *n* = 1^a^		NDM; *n* = 11^a^				KPC; *n* = 9^a^		OXA-48; *n* = 6^a^				c-np^b^; *n* = 10^a^			
		+	+/−	−	NG (NI)	+	NG	+	+/−	−	NG (NI)	+	NG	+	+/−	−	NG	+	+/−	−	NG (NI)
Columbia blood agar	BD	7				1		10			(1)	9		6						10	
chromID CPS agar	bioMérieux	7				1		11				9		6						10	
CHROMagar Orientation Medium	BD	7				1		11				9		6					1	9	
Brilliance UTI agar	Oxoid	7				1		11				9		5	1				3	7	
Brilliance UTI Clarity agar	Oxoid	7				1		11				9		5	1				3	7	
Brilliance ESBL agar	Oxoid	6			(1)	1		10			(1)	9		3		1	2		1	9	
chromID ESBL agar	bioMérieux	7				1		11				9		5			1			9	1
Brilliance CRE agar	Oxoid	6			1	1		11				8	1	2	1		3		2	8	
chromID CARBA agar	bioMérieux	3	2	2		1		10	1			9		5	1					7	3
KPC CHROM agar	GRASO	7				1		11				9		5		1		2	1	6	1
chromID OXA-48 agar	bioMérieux				7		1	1			10		9	6							10
MH	BD	7				1		10		1		9		6					2	7	(1)
MHE	bioMérieux	7				1		10		1		9		6						8	(2)
MHF	bioMérieux	1		6		1		2	3	6		9		6						10	
MH	Oxoid	7				1		11				9		5	1					3	(7)
MH	GRASO	7				1		11				9		6				1		6	(3)
MH	Liofilchem	7				1		10	1			9		6						9	(1)
MH	Bio-Rad	5		2		1		2	3	6		9		6				1	1	8	

^a^+, positive; +/−, questionable; −, negative; (NI), not interpretable; NG, no growth

^b^c-np, carbapenemase-non-producers

**Table 3:** The Carba NP results for *Pseudomonas* spp. (15 carbapenemase producers and 6 carbapenemase non-producers) and the CarbAcineto results for *Acinetobacter* spp. (32 carbapenemase producers and 10 carbapenemase non-producers) grown on a set of microbiological media

		Carba NP										CarbAcineto											
		*Pseudomonas* spp.										*Acinetobacter* spp.											
Microbiological media	Manufacturer	VIM; *n* = 14^a^				IMP; *n* = 1^a^		c-np^b^; *n* = 6^a^				VIM; *n* = 16^a^				OXA-like; *n* = 16^a^				c-np^b^; *n* = 10^a^			
		+	+/−	−	NG (NI)	+	NG (NI)	+	+/−	−	NG (NI)	+	+/−	−	NG (NI)	+	+/−	−	NG (NI)	+	+/−	−	NG (NI)
Columbia blood agar	BD	14				1				6		16				16						10	
chromID CPS agar	bioMérieux	14				1				6		14	2			16						9	(1)
CHROMagar Orientation Medium	BD	13	1			1			2	4		11	4	1		15		1			1	9	
Brilliance UTI agar	Oxoid	14				1			3	3		12			4	15		1				9	(1)
Brilliance UTI Clarity agar	Oxoid	12	1		(1)	1		1	2	3		14			2	15	1				1	8	(1)
Brilliance ESBL agar	Oxoid	8	3	2	(1)	1		1		5					13 (3)	13	2		1		1	2	6 (1)
chromID ESBL agar	bioMérieux	12	1	1		1			1	4	1	14	1	1		15	1					8	1 (1)
Brilliance CRE agar	Oxoid	14				1		1	3	2		10	2		4	15	1					7	2 (1)
chromID CARBA agar	bioMérieux	10	1	3		1		1	1	3	1	10			6	15	1					8	2
KPC CHROM agar	GRASO	12		2		1				6		11	2	3		15		1				8	2
chromID OXA-48 agar	bioMérieux	3		3	8		1			3	3		1	9	6	9	2	4	1			3	7
MH	Bio-Rad	9	5			1		1		5		16				16				1	1	8	
MH	Liofilchem	13	1			1			1	5		16				14	1	1				10	
MH	GRASO	13	1			1				6		16				16					1	8	(1)
MH	BD	14				1				6		16				15	1			1	2	7	
MH	Oxoid	13		1		1				6		16				16						10	
MHE	bioMérieux	14				1			2	4		16				16						9	(1)
MHF	bioMérieux	5	3	6		1				6		11	3	2		15	1				1	8	(1)

^a^+, positive; +/−, questionable; −, negative; (NI), not interpretable; NG, no growth

^b^c-np, carbapenemase-non-producers

### The Carba NP and CarbAcineto tests in routine reference diagnostics

In the second part of this work, the Carba NP and CarbAcineto tests were implemented into the routine activity of the NRCST, which is responsible for the reference diagnostics and surveillance of carbapenemase-producing Gram-negative pathogens in Poland. Both tests were performed along with the phenylboronic acid disc test [27], double-disc synergy test with EDTA (DDST-EDTA) [28], temocillin disc test [29] for the phenotypic detection of KPCs, MBLs and OXA-48 types, respectively [30], spectrophotometric assay for imipenem hydrolysis [31] and PCR for *bla*_KPC_-, *bla*_VIM_-, *bla*_IMP_-, *bla*_NDM_-, *bla*_OXA-48_-, *bla*_OXA-23_-, *bla*_OXA-24/40_- and *bla*_OXA-58_-like genes [32]. This analysis was done on 1282 isolates in total (Table 4), including 915 Enterobacteriaceae, 309 *Pseudomonas* spp. and 58 *Acinetobacter* spp. collected from April to the end of December 2014 in hospitals all over Poland. All these isolates were cultivated only on Columbia blood agar plates.

**Table 4:** Performance of the Carba NP/CarbAcineto tests in the analysis of 1282 surveillance isolates submitted to the National Reference Centre for Susceptibility Testing (NRCST) from April to December 2014

Enterobacteriaceae (total, *n* = 915)^a^	*Pseudomonas* spp. (total, *n* = 309)^a^	*Acinetobacter* spp. (total, *n* = 58)^a^
Carbapenemase-positive, *n* = 451	Carbapenemase-positive, *n* = 118	Carbapenemase-positive, *n* = 54
Carba NP positive, *n* = 432	Carba NP positive, *n* = 115	CarbAcineto positive, *n* = 48
KPC, *n* = 77	VIM, *n* = 115	*A. baumannii* VIM, *n* = 2
*K. pneumoniae*, *n* = 75	*P. aeruginosa*, *n* = 111	*A. lwoffii* VIM, *n* = 3
*E. coli*, *n* = 1	*P. putida*, *n* = 4	*A. junii* VIM, *n* = 3
*E. cloacae*, *n* = 1		*A. baumannii* OXA-24/40-like, *n* = 30
NDM, *n* = 275		*A. baumannii* OXA-23-like, *n* = 10
*K. pneumoniae*, *n* = 267		
*E. coli*, *n* = 2		
*E. cloacae*, *n* = 4		
*S. marcescens*, *n* = 2		
VIM, *n* = 57		
*K. pneumoniae*, *n* = 12		
*E. coli*, *n* = 6		
*E. cloacae*, *n* = 26		
*C. freundii*, *n* = 8		
*S. marcescens*, *n* = 4		
*K. oxytoca*, *n* = 1		
OXA-48, *n* = 23		
*K. pneumoniae*, *n* = 7		
*E. coli*, *n* = 14		
*E. aerogenes*, *n* = 2		
Carba NP questionable, *n* = 18	Carba NP questionable, *n* = 3	CarbAcineto questionable, *n* = 6
*K. pneumoniae* NDM, *n* = 14	*P. aeruginosa* VIM, *n* = 3	*A. baumannii* OXA-24/40-like, *n* = 1
*K. pneumoniae* OXA-48, *n* = 1		*A. baumannii* OXA-23-like, *n* = 3
*E. coli* OXA-48, *n* = 1		*A. baumannii* OXA-58, *n* = 2
*E. cloacae* KPC, *n* = 1		
*C. freundii* VIM, *n* = 1		
Carba NP not interpretable, *n* = 1		
*K. pneumoniae* NDM, *n* = 1		
Carbapenemase-negative, *n* = 464	Carbapenemase-negative, *n* = 191	Carbapenemase-negative, *n* = 4
Carba NP negative, *n* = 433	Carba NP negative, *n* = 189	CarbAcineto negative, *n* = 4
Carba NP questionable, *n* = 30	Carba NP questionable, *n* = 2	
*K. pneumoniae*, *n* = 19	*P. aeruginosa*, *n* = 2	
*E. cloacae*, *n* = 9		
*S. marcescens*, *n* = 1		
*P. mirabilis, n* = 1		
Carba NP not interpretable, *n* = 1		
*K. pneumoniae*, *n* = 1		
Sensitivity 95.8% (95% CI, 93.9–97.6%)	Sensitivity 97.5% (95% CI, 94.6–100%)	Sensitivity 88.9% (95% CI, 80.4–97.4%)
Specificity 93.3% (95% CI, 90.9–95.6%)	Specificity 99.0% (95% CI, 97.6–100%)	
PPV 95.8% (95% CI, 93.9–97.6%)	PPV 97.5% (95% CI, 94.6–100%)	PPV 88.9% (95% CI, 80.4–97.4%)
NPV 93.3% (95% CI, 90.9–95.6%)	NPV 99.0% (95% CI, 97.6–100%)	

^a^PPV, positive predictive value; NPV, negative predictive value; CI, confidence interval

## Results

### Assessment of the Carba NP and CarbAcineto tests and the cultivation media

For Enterobacteriaceae, the Carba NP test performed well, with the carbapenemase-producing strains cultured on most of the non-selective or screening media (Table 2). False-negative results were obtained only for single isolates with VIMs or OXA-48s grown on three types of chromogenic media. When bacteria were cultivated on Mueller–Hinton (MH) agars, the Carba NP test was positive for all KPC, IMP and all but one OXA-48 producers, while false-negative or questionable results were obtained for NDM- and VIM-producing strains. For a number of carbapenemase-negative strains picked up from some media, the results were questionable and for single strains were false-positive.

The results of the Carba NP test for *Pseudomonas* spp. strains are presented in Table 3. In the case of carbapenemase (VIM or IMP) producers, the best results were observed for the strains cultivated on the Columbia blood agar, chromID CPS, Brilliance UTI agar (Oxoid) and Brilliance CRE agar (Oxoid). The false-negative results were observed for some isolates cultivated on five other chromogenic media, and on MH (Oxoid) and MHF (bioMérieux) agars. For single carbapenemase-negative strains cultivated on five media, false-positive results were observed.

The results of the CarbAcineto test for *Acinetobacter* spp. strains are shown in Table 3. As in the case of Enterobacteriaceae and *Pseudomonas* spp., the impact of the cultivation medium on CarbAcineto was revealed; moreover, for isolates with OXA-like carbapenemases, the correct results were observed more often than for VIM-positive strains (Table 3).

### The Carba NP and CarbAcineto tests in routine reference diagnostics

The results of the comparison of the Carba NP/CarbAcineto tests with the standard methodology used by the NRCST, obtained with 1282 bacterial isolates suspected of carbapenemase production, are presented in Table 4. The phenotypic tests and PCRs detected various carbapenemases in 451/915 Enterobacteriaceae, 118/309 *Pseudomonas* spp. and 54/58 *Acinetobacter* spp. isolates. For Enterobacteriaceae, the overall sensitivity and positive predictive value (PPV) of Carba NP were 95.8% [95% confidence interval (CI), 93.9–97.6%], and the specificity and negative predictive value (NPV) were 93.3% (95% CI, 90.9–95.6%). For *Pseudomonas* spp. the sensitivity and PPV were 97.5% (95% CI, 94.6–100%), and the specificity and NPV were 99.0% (95% CI, 97.6–100%). For *Acinetobacter* spp., the sensitivity and PPV of CarbAcineto were 88.9% (95% CI, 80.4–97.4%).

## Discussion

The Carba NP test was described for the first time in 2012 by Nordmann et al. as a rapid, easy and cheap method for carbapenemase detection in Enterobacteriaceae and *Pseudomonas* spp., with high sensitivity and specificity [1, 15]. In previous studies, the test was evaluated by several groups, often confirming the original observations [2, 19, 33–35]. However, one study undermined the sensitivity in the case of MBL-producing strains [2]. The problem turned out to be associated with the cultivation medium used [16–18], and the follow-up study by Dortet et al. documented well the impact of microbiological media on the performance of Carba NP [18].

In the first part of this work, well-characterised Enterobacteriaceae, *Pseudomonas* spp. and *Acinetobacter* spp. strains with various carbapenemases were used, along with a broad panel of commercially available media. The results clearly illustrated the influence of the media on the Carba NP/CarbAcineto results. According to our experience, the Columbia blood agar is the best choice for the cultivation of bacteria for the Carba NP/CarbAcineto tests, providing the highest sensitivity and clarity of the results. The VIM or NDM producers harvested from most of the MH agars yielded a remarkable number of false-negative results, which were most probably related to lower zinc ions’ concentrations that is crucial for the MBL activity [18]. Owing to the low incidence of OXA-48 producers in Poland so far, only six such isolates were used in this work, and all of these were clearly positive in Carba NP, in contrast to some other reports [17, 25, 34–37]. This study has definitely confirmed the usefulness of the CarbAcineto test for the detection of the acquired carbapenemases in *Acinetobacter* spp. [8, 38, 39] and for distinguishing such isolates from hyperproducers of the natural OXA-51 types and from the carbapenemase-negative carbapenem-non-susceptible isolates, which is important for epidemiological reasons.

The Carba NP and CarbAcineto tests have been used in the NRCST from the end of 2013 and March 2014, respectively, and now are included in its routine algorithm for carbapenemase detection as reliable, inexpensive and easy screening tests, reducing remarkably the time for the first feedback information for clinical laboratories. Based on own experience, and in the context of the alarming spread of carbapenemase-producing Gram-negative pathogens in Poland, the NRCST has been recommending Carba NP/CarbAcineto tests for use in diagnostic microbiology laboratories all over the country.
